# Neonatal Oxidative Stress Impairs Cortical Synapse Formation and GABA Homeostasis in Parvalbumin-Expressing Interneurons

**DOI:** 10.1155/2022/8469756

**Published:** 2022-05-25

**Authors:** Till Scheuer, Stefanie Endesfelder, Elena auf dem Brinke, Christoph Bührer, Thomas Schmitz

**Affiliations:** Charité-Universitätsmedizin Berlin, Corporate Member of Freie Universität Berlin and Humboldt Universität zu Berlin, Department of Neonatology, Augustenburger Platz 1, 13353 Berlin, Germany

## Abstract

Neonatal brain injury is often caused by preterm birth. Brain development is vulnerable to increased environmental stress, including oxidative stress challenges. Due to a premature change of the fetal living environment from low oxygen *in utero* into postnatal high-oxygen room air conditions *ex utero*, the immature preterm brain is exposed to a relative hyperoxia, which can induce oxidative stress and impair neuronal cell development. To simulate the drastic increase of oxygen exposure in the immature brain, 5-day-old C57BL/6 mice were exposed to hyperoxia (80% oxygen) for 48 hours or kept in room air (normoxia, 21% oxygen) and mice were analyzed for maturational alterations of cortical GABAergic interneurons. As a result, oxidative stress was indicated by elevated tyrosine nitration of proteins. We found perturbation of perineuronal net formation in line with decreased density of parvalbumin-expressing (PVALB) cortical interneurons in hyperoxic mice. Moreover, maturational deficits of cortical PVALB+ interneurons were obtained by decreased glutamate decarboxylase 67 (GAD67) protein expression in Western blot analysis and lower gamma-aminobutyric acid (GABA) fluorescence intensity in immunostaining. Hyperoxia-induced oxidative stress affected cortical synaptogenesis by decreasing *synapsin 1*, *synapsin 2*, and *synaptophysin* expression. Developmental delay of synaptic marker expression was demonstrated together with decreased PI3K-signaling as a pathway being involved in synaptogenesis. These results elucidate that neonatal oxidative stress caused by increased oxygen exposure can lead to GABAergic interneuron damage which may serve as an explanation for the high incidence of psychiatric and behavioral alterations found in preterm infants.

## 1. Introduction

Oxygen availability is essential for all aerobic organisms. Nevertheless, increased oxygen levels can induce oxidative stress, which is a high risk factor for oxidative damage [1, 2].

Soon after human birth, arterial oxygen tension increases from 25 mmHg up to 65-80 mmHg, even without extra oxygen supply [3]. This 3-to 4-fold increase of oxygen disposability as compared to the fetal (i.e., prenatal) situation hits the immature brain of preterm infants in a developmental phase of exponential precursor cell expansion and neuronal maturation [4, 5]. In general, preterm infants are susceptible to injury caused by oxidative stress. The immature enzymatic oxidative stress defense system is uncapable to neutralize the increased formation of reactive oxygen species [6–8]. Therefore, oxidative stress can damage DNA, proteins, and lipids as a mechanism of cellular damage and can lead to necrotic and apoptotic cell death [7, 9]. Oxygen may also function as a distinct regulator of brain development by decreasing cell proliferation and inhibiting oxygen-sensitive signaling [10–12]. Multiple cell types can be affected [6, 7]; for instance, oxidative stress can cause dysmaturation in immature oligodendroglia with hypomyelination as a consequence [13, 14], as well as cellular damage in GABAergic interneurons, leading to decreased neuronal activity [15]. A loss of neurons and glial cells can cause several neurological diseases [16]. During late gestation and early postnatal life, the gamma aminobutyric acid (GABA) neurotransmitter system undergoes rapid maturation [17]. Between gestational weeks 16 and 35, generation and migration of GABAergic interneurons peak in humans [18, 19]. A profound vulnerability of GABAergic interneurons during this critical phase of development to external factors has been highlighted in human studies to be relevant after prenatal stress [20], perinatal inflammation [21], and premature birth [22, 23]. GABAergic interneurons are characterized by heterogeneous subpopulations with subtype-specific morphology, electrophysiological properties, molecular features, and marker expression [24, 25]. Cortical interneurons immunoreactive for parvalbumin (PVALB) constitute the largest interneuronal population in the mammalian cortex. Premature birth and increased oxidative stress have been reported to cause developmental changes of PVALB+ interneurons [23, 26]. Developmental alterations of PVALB+ interneurons might be the cause of various psychiatric disabilities [27, 28].

In our study, we are using a neonatal oxidative stress model of preterm birth brain injury, in which five-day-old (P5) mouse pups are exposed to hyperoxia (80% oxygen) for 48 hours, causing a 3- to 4-fold increase of oxygen levels [13, 29]. In a previous study, we could demonstrate a significant reduction of cortical PVALB+ interneurons, both acutely and during further developmental until adulthood, which was accompanied by altered behavior being caused by neonatal hyperoxia/oxidative stress exposure [30]. Here, we aim to define the impact of neonatal oxidative stress on GABA homeostasis and synapse formation as key aspects for functional maturation of cortical PVALB+ interneurons playing a role in psychiatric disorders often diagnosed in preterm infants.

## 2. Methods

### 2.1. Animal Experiments

For our animal experiments, we had received permission by the Animal Welfare Committee of Berlin. All experimental procedures moreover followed institutional guidelines and also ARRIVE guidelines. Exposure to 80% oxygen was performed using an OxyCycler chamber (OxyCycler, BioSpherix, Lacona, NY, USA) in which wild-type mice (C57BL/6) were put starting at age P5 together with their breeding mothers. After being taken out of the chamber at age P7, newborn mice recovered in room air for different time spans until ages P9, P11, P15, and P30, for further analysis. All animals were obtained from Janvier Labs and housed under environment-controlled conditions with a constant 12 h/12 h light/dark cycle, ambient temperature, and relative humidity of 60% with *ad libitum* access to the same food and water.

### 2.2. Real-Time PCR

As previously described [30], total cortical RNA was isolated by acidic phenol/chloroform extraction (peqGold RNAPure, PEQLAB Biotechnologie, Erlangen, Germany) from mouse cortical samples. After DNase (Qiagen, Hilden, Germany) pretreatment, two *μ*g of total RNA was reverse transcribed (M-MLV Reverse Transcriptase, Promega, Walldorf, Germany) for cDNA synthesis. Specific primers were used for determination of gene expression of interneuronal maturation markers (immature: *Gabra3*, *Gabra5*; mature: *Gabra1*, *Gabra4*) and synapsis markers *Syn1*, *Syn2*, *Syp*, *Syt1*, and *Syt2* (Table 1) using SyGreen Mix Hi-ROX (NIPPON Genetics Europe, Düren, Germany). The expression was analyzed with the StepOnePlus™ Real-Time PCR System (Applied Biosystems, Life Technologies, Carlsbad, CA) according to the 2^−*ΔΔ*CT^ method [31]. For internal reference, housekeeping gene *Hprt* was used.

### 2.3. Western Blot

A fixed amount of 20 *μ*g of isolated protein from mouse cortex was separated by SDS-PAGE using a 4–20% Criterion™ TGX™ Precast Mini/Midi Protein Gel (Bio-Rad, Feldkirchen, Germany). As described elsewhere [30], blotting (Trans-Blot Turbo Transfer System, Bio-Rad) was performed to a nitrocellulose membrane. Unspecific binding sites were blocked (Roti-Block, Car-Roth, Karlsruhe, Germany), and membranes were thereafter incubated with primary antibodies as follows: anti-nitrotyrosine (Millipore, 06-284, 1 : 1000), anti-GAD1/GAD67 (Novus Biologicals, NBP102161, 1 : 1000), anti-phosphoinositide 3-kinase (Upstate, 06-497, 1 : 1000), anti-phospho-AKT (Cell Signaling Technologies, 9271, 1 : 500), and anti-*β*-AKTIN (Sigma, A5316, 1 : 5000), followed by appropriate secondary antibodies conjugated with horseradish peroxidase (donkey anti-rabbit Pierce #31458, 1 : 5000; rabbit anti-mouse Dako P0260, 1 : 5000; goat anti-chicken Invitrogen A16054, 1 : 5000) and detection by chemiluminescence (PerkinElmer, USA). Loading control *β*-actin was used as an internal reference [30].

### 2.4. Immunohistochemistry

Anesthetized mice were transcardially perfused first with phosphate-buffered saline (PBS) and then again with 4% paraformaldehyde (PFA) solution. After dissection, brains were furthermore fixed in 4% PFA at 4°C overnight as previously described [30]. Washing was performed using PBS, followed by cryoprotection in sucrose solutions at 4°C, starting with 5% sucrose dissolved in PBS for one hour, then 10% sucrose solution overnight, and then with 30% sucrose solution until the organs dropped to the bottom of the glass. A cryotome (Thermo Fisher Scientific) was used to produce coronal brain sections of 10 *μ*m thickness to be stored on slides at -20°C. For fluorescence staining, the following antibodies were used: rabbit anti-parvalbumin (PVALB, Abcam ab11427, 1 : 1000), mouse anti-PVALB (Swant, PV235, 1 : 1000), rabbit anti-synapsin 1/2 (Synaptic Systems, 106002, 1 : 400), and mouse anti-GABA (Abcam, ab86186, 1 : 100). After incubation with the primary antibodies, brain sections were incubated with appropriate secondary antibodies Alexa Fluor 594 goat anti-rabbit IgG (Jackson ImmunoResearch, 111-585-003, 1 : 200) and Alexa Fluor 488 goat anti-mouse IgG (Jackson ImmunoResearch, 115-545-003, 1 : 200). Biotin-conjugated lectin from *Wisteria floribunda* (Sigma-Aldrich, L1516, 1 : 200) and ExtrAvidin®–FITC (Sigma-Aldrich, E2761, 1 : 200) were served for perineuronal net staining. Incubation of primary antibodies occurred in antibody diluent (DAKO, S3022) at 4°C overnight. Incubation of secondary antibodies occurred in antibody diluent (DAKO, S3022) at room temperature for 1 hour. After a final washing step, mounting of sections was performed in Fluoroshield with DAPI (4′, 6-diamidino-2-phenylindole, Sigma).

### 2.5. Microscope Measurements

Keyence compact fluorescent microscope BZ 9000 with a 10x, 20x, and 40x objective; the BZ-II Viewer software; and BZ-II Analyzer software (Keyence, Osaka, Japan) were used for analysis of immunohistochemically stained brain sections. Quantification of immunolabeled cells was performed with the help of Photoshop CSM (Adobe) software under minimal adjustment of contrast.

### 2.6. Statistics

For presentation of results in figures, boxplots were generated with median, minimum, maximum, upper, and lower quartile for the distinct experimental groups. All data sets were analyzed for significant outliers. Due to normal distribution, *t*-test was used (two-tailed) for statistical analysis. GraphPad Prism 5.0 software (GraphPad Software, USA) was used to generate all graphics and calculate statistics. As for all statistical analyses, *n* refers to the number of analyzed animals.

## 3. Results

### 3.1. Neonatal Hyperoxia Initiates Oxidative Stress Cortical Samples

**Figure 1:**
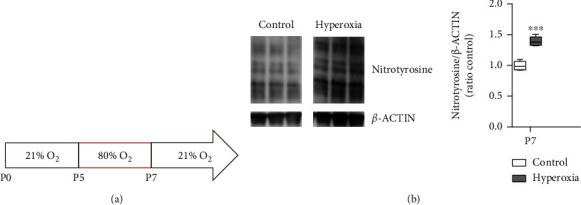
Oxidative stress in the immature cortex of mice exposed to neonatal hyperoxia. Schematic overview of oxidative stress exposure (a). Five-day-old mouse pups were exposed to 80% oxygen for 48 hours and kept at room air conditions (21% oxygen) thereafter. Western blot analysis of nitrotyrosine reveals increased oxidative stress-induced protein nitration after exposure to neonatal hyperoxia at P7 (b) (*n* = 6, *t*-test ^∗∗∗^*P* < 0.001).

Neonatal hyperoxia from P5 to P7 (Figure 1(a)) induces oxidative stress (OS) in mouse brains previously confirmed by increased lipid peroxidation [30]. OS can alter brain development and inhibits neuronal development in different brain regions [30, 32, 33]. To verify oxidative stress on protein level, we analyzed tyrosine nitration by Western blot at P7. Moreover, increased tyrosine nitration of proteins can alter protein function [34]. Nitrotyrosine Western blot indicates cortical OS by increased tyrosine nitration of proteins after exposure to neonatal hyperoxia from P5 to P7 (Figure 1(b)).

### 3.2. Altered Cortical Perineuronal Net Formation in Response to Postnatal OS

**Figure 2:**
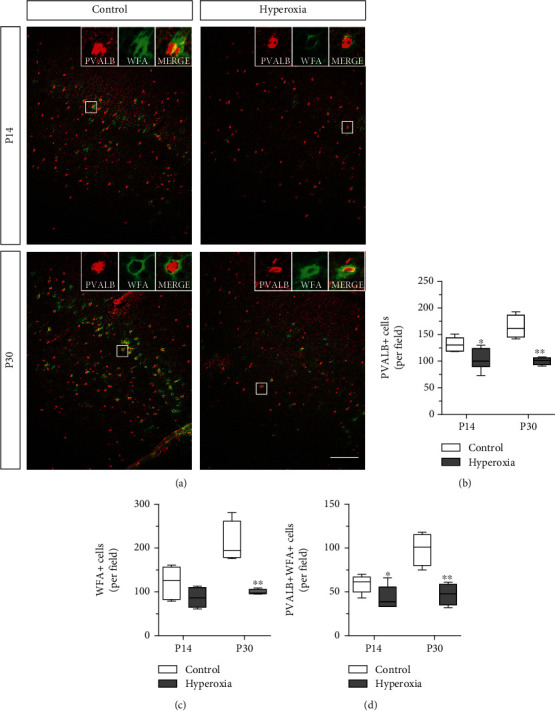
Perineuronal net formation of cortical PVALB+ interneurons. Immunohistochemical staining for parvalbumin (PVALB) and *Wisteria floribunda agglutinin* (WFA) of 10 *μ*m coronal brain sections obtained from mice after 48 h neonatal hyperoxia (P5-P7) or controls at the ages P14 and P30 (a). The number of PVALB+ interneurons was consistently reduced after exposure to neonatal hyperoxia compared to control (b). At P14, the number of WFA+ cells was not significantly affected by hyperoxia exposure. At P30, formation of WFA+ perineuronal nets was significantly reduced in the cortex of animals previously exposed to hyperoxia compared to control animals (c). In addition, the number of WFA+ and PVALB+ interneurons was reduced at both time points in mice previously exposed to hyperoxia (d). Scale bar 100 *μ*m (P14 *n* = 6 and P30 *n* = 4, *t*-test, ^∗^*P* < 0.05 and ^∗∗^*P* < 0.01).

Function of GABAergic interneurons is highly regulated by perineuronal net (PNN) formation. The PNN is an extracellular matrix assembly preferentially ensheathing parvalbumin-expressing (PVALB+) interneurons [35, 36]. Cortical PNNs first appear around P14 [37]. Perineuronal nets are commonly labeled with the plant lectin *Wisteria floribunda agglutinin* (WFA) [38]. To investigate the impact of OS induced by hyperoxia from P5 to P7 on developmental PNN formation, we analyzed the numbers of cortical WFA+ cells and of WFA+ PVALB+ colabeled cells by immunohistochemistry at ages P14 and P30. In line with a previous study [30], we observed a decreased density of cortical PVALB-expressing interneurons after exposure to OS at both ages (Figures 2(a) and 2(b). The number of WFA+ cells was not significantly affected by OS at P14. Additionally, WFA pixel intensity was variable in both groups at this time point (Figures 2(a) and 2(c)). At P30, the number of WFA+ cells was significantly reduced in mice after postnatal OS (Figures 2(a) and 2(c)). At both time points, the number of PVALB+ WFA+ colabeled interneurons was reduced in cortices of the hyperoxia/OS group as compared to controls (Figures 2(a) and 2(d)), indicating alteration of PNN formation and possible functional and maturational impairments of PVALB+ interneurons.

### 3.3. GABA Receptor Alpha Subunit Expression Does Not Indicate Maturational Impairment of Cortical Interneurons

**Figure 3:**
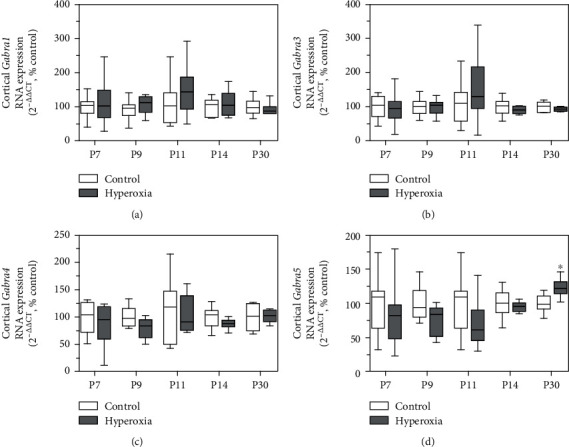
Maturation of cortical GABAergic interneurons. GABA receptor alpha subunit distribution indicates maturational state of GABAergic interneurons. *Gabra3* and *Gabra5* expression indicates immature state. *Gabra1* and *Gabra4* expression indicates mature state. AT P7, P9, P11, P14, and P30, qPCR analysis of *Gabra1* (a), *Gabra3* (b), *Gabra4* (c), and *Gabra5* (d) did not indicate maturational differences of GABAergic interneurons. However, at P30 RNA, expression of *Gabra5* was increased in cortical samples of mice previously exposed to hyperoxia (*n* = 6-8, *t*-test ^∗^*P* < 0.05).

Maturation of GABAergic interneurons occurs until P20-30 in rodents [39]. More mature stages of interneurons can be characterized by a high level of glutamate decarboxylase (GAD) activity, high GABA concentrations, increased activity of GABA-transporters, and a specific distribution of GABA receptor alpha subunits (mature = expression of receptor subunits *Gabra*1 and *Gabra*4, immature = expression of *Gabra*3 and *Gabra*5 subunits) [39]. In theory, maturational delay in our studies may appear in connection with delayed or reduced perineuronal net formation. In order to investigate a potential perturbation of GABAergic interneuronal maturation, we analyzed cortical gene expression of *Gabra* subunits at the ages P7, P9, P11, P14, and P30 by qPCR. As a result, cortical RNA expression levels of *Gabra1*, *Gabra3*, *Gabra4*, and *Gabra5* were not affected by neonatal hyperoxia/OS in mice analyzed at ages P7, P9, P11, and P14 (Figures 3(a)–3(d)). At P30, an increased expression of *Gabra5* in cortical samples of the hyperoxia group was observed and might indicate increased immaturity of cortical GABAergic interneurons (Figure 3(d)). However, RNA expression of the other *Gabra* subunits was not affected by hyperoxia/OS at this time point, hence indicating no robust effect of neonatal OS on interneuronal maturation. It cannot be excluded, though, that detection of maturational changes in PVALB+ interneurons can be masked by *Gabra* subunit gene expression of other cortical interneuron subtypes being unaffected by OS [30].

### 3.4. Neonatal Oxidative Stress Affects GABA Synthesis of PVALB+ Interneurons

**Figure 4:**
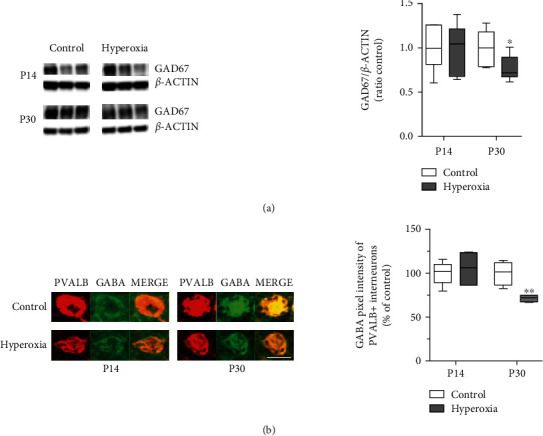
GABA synthesis of cortical PVALB+ interneurons. Protein expression analysis of the 67 kDa glutamate decarboxylase isoform (GAD67) at P14 and P30 (a) indicates decreased GAD67 expression in animals exposed to neonatal hyperoxia at the age P30 (for Western blot: *n* = 6, *t*-test ^∗^*P* < 0.05). Immunohistochemical staining for parvalbumin (PVALB) and gamma-aminobutyric acid (GABA) of 10 *μ*m coronal brain sections at the ages P14 and P30 (b) reveals decreased GABA intensity of PVALB+ cortical interneurons in the hyperoxia group at P30 (for immunohistochemistry, P14 *n* = 6 and P30 *n* = 4, *t*-test, ^∗∗^*P* < 0.01). Scale bar 10 *μ*m.

High expression activity of glutamate decarboxylase (GAD) is specific for mature GABAergic interneurons [39]. GAD65 and GAD67 are two distinct isoforms synthesizing the inhibitory neurotransmitter gamma-aminobutyric acid (GABA) [40]. Due to the fact that the expression of the GAD65 isoform in cortical PVALB+ interneurons is relatively low in comparison to other interneuron subtypes and that protein expression of the GAD67 isoform is closely related to overall GABA levels [41, 42], we selectively analyzed cortical GAD67 protein expression in hyperoxia/OS mice and in normoxia control mice by Western blot at the ages P14 and P30. As a result, cortical GAD67 protein expression was identical in both experimental groups at P14. In contrast, a significant reduction of GAD67 protein expression was observed in cortical protein samples of P30 OS mice as compared to control mice (Figure 4(a)). To evaluate decreased GAD67 expression on GABA levels of cortical PVALB+ interneurons, we performed immunostaining for GABA and PVALB at the ages P14 and P30. In correspondence with the reduction of GAD67 protein expression, GABA intensity of cortical PVALB+ interneurons was significantly diminished in hyperoxia animals at P30 (Figure 4(b)). These results strongly point towards a possible functional impairment and maturational delay of cortical PVALB+ GABAergic interneurons caused by neonatal OS.

### 3.5. Altered Cortical Synaptogenesis after Exposure to Neonatal Oxidative Stress

**Figure 5:**
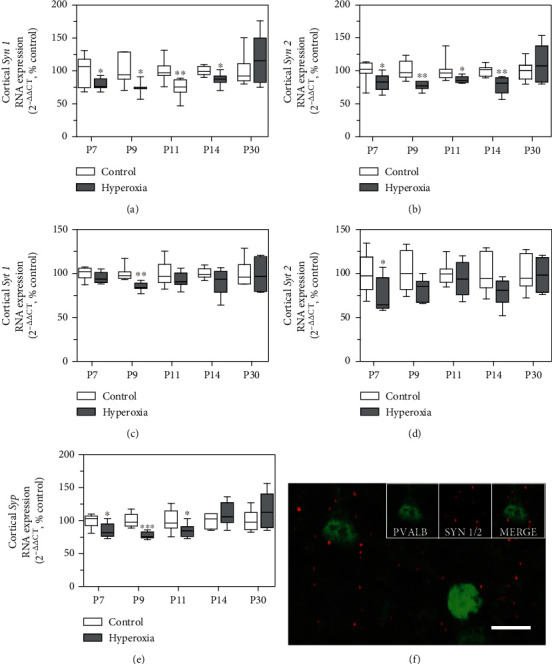
Delayed expression of synaptic markers after exposure to neonatal hyperoxia. Real-time PCR expression analysis of the synaptic markers: synapsin 1 (*Syn1*), synapsin 2 (*Syn2*), synaptotagmin 1 (*Syt1*), synaptotagmin2 (*Syt2*), and synaptophysin (*Syp*) at the age P7, P9, P11, P14, and P30. Gene expression of *Syn1* was decreased in cortical samples of animals previously exposed to neonatal hyperoxia (P5-P7) at the ages P7, P9, P11, and P14 and returns to control level at P30 (a). At P7-P14, cortical RNA expression of *Syn2* was decreased in hyperoxia animals compared to control litters. At P30, *Syn2* expression returns to control level (b). RNA expression of *Syt1* was reduced at P9 (c) in the hyperoxia group. Cortical expression of *Syt2* was reduced at P11 in mice previously exposed to hyperoxia and returns to control level at P14 (d). At the ages P7, P9, and P11, cortical RNA expression of *Syp* was decreased in the hyperoxia group (e) (for qPCR: *n* = 6-8, *t*-test, ^∗^*P* < 0.05; ^∗∗^*P* < 0.01; ^∗∗∗^*P* < 0.001). Immunohistochemical staining for parvalbumin (PVALB) and synapsin 1/2 (SYN1/2) of 10 *μ*m coronal brain sections at the age P14 reveals SYN1/2 expression of cortical PVALB+ interneurons (f). Scale bar 20 *μ*m.

In the cortex, synaptogenesis during pregnancy peaks in the third trimester [43, 44]. Disrupted synaptic development has been observed in other preterm birth brain injury models [45], and oxidative stress affects synaptic plasticity [46]. The synaptic proteins synapsin 1 (SYN1), synapsin 2 (SYN2), synaptotagmin 1 (SYT1), synaptotagmin 2 (SYT2), and synaptophysin (SYP) are involved in neurotransmitter release of parvalbumin-expressing GABAergic interneurons [47–50]. To investigate the impact of neonatal hyperoxia exposure from P5 to P7 on cortical synapses, we analyzed the RNA expression of *Syn1*, *Syn2*, *Syt1*, *Syt2*, and *Syp* in cortical samples by qPCR at the ages P7, P9, P11, P14, and P30. Cortical gene expression of *Syn1* and *Syn2* was significantly reduced at P7, P9, P11, and P14 and returned to control level at P30 in mice exposed to hyperoxia/OS (Figures 5(a) and 5(b)). Immunohistochemistry for PVALB and SYN1/2 revealed SYN1/2 expression of PVALB+ interneurons (Figure 5(f)). However, RNA expression of *Syt1* and *Syt2* was less affected by hyperoxia/OS. Syt1 expression was significantly reduced at P9, and *Syt2* expression was lower at P7 (Figures 5(c) and 5(d)). RNA expression of *Syp*, a synaptic marker for interneuronal crosstalk, was reduced at P7, P9, and P11 after neonatal hyperoxia/OS (Figure 5(e)). As a consequence, OS may inhibit neurotransmitter release of GABAergic PVALB+ interneurons by impaired regulation of vesicle trafficking as one possible mechanism, since it is highly regulated by synapsins [51].

### 3.6. Neonatal OS Decreases Cortical PI3K Activity

**Figure 6:**
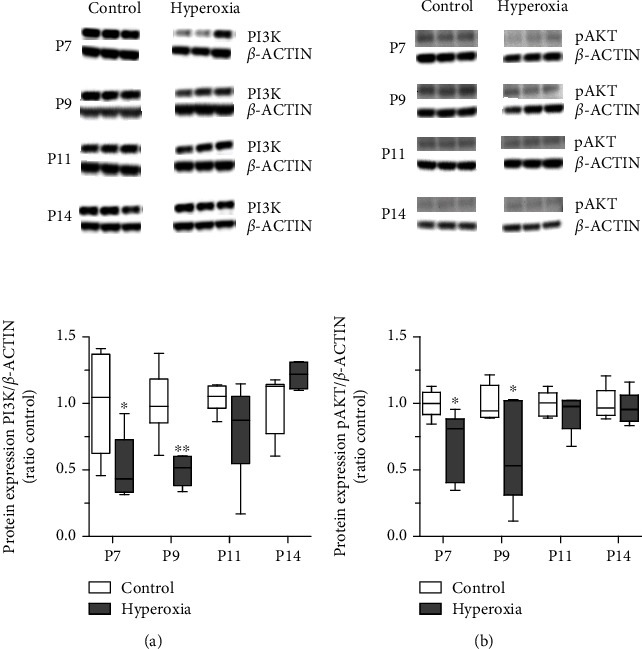
Phosphoinositide 3-kinase signaling in cortical brain samples after exposure to hyperoxia. Protein expression analysis of phosphoinositide 3-kinases (PI3K) and its downstream target phospho-Akt (pAKT) by Western blot indicates decreased expression of PI3K (a) and pAKT (b) at the ages P7 and P9 in cortical samples of mice exposed to hyperoxia compared to control (*n* = 6, *t*-test ^∗^*P* < 0.05 and ^∗∗^*P* < 0.01).

Phosphoinositide 3-kinase (PI3K) activity is essential for various functions of brain development such as cellular growth, migration, differentiation, and survival [52]. In several studies, PI3K function has been investigated for synaptogenesis and dendritic arborization [53–55] and also interneuronal survival [56]. Therefore, we analyzed the cortical PI3K-Akt signaling pathway by Western blot for PI3K and phosphor-AKT (pAKT) expression at ages P7, P9, P11, and P14. Protein expression of PI3K and pAKT was significantly reduced after neonatal OS at P7 and also after two days of recovery at P9 as compared to control animals (Figures 6(a) and 6(b)). At P11 and P14, protein expression of PI3K and pAKT returns to control levels (Figures 5(a) and 5(b)), indicating recovery of the PI3K-Akt signaling pathway after prior exposure to hyperoxia/OS.

## 4. Discussion

GABA- (gamma-aminobutyric acid-) ergic interneurons represent about 25-30% of all cortical neuronal cells [57]. In some cortical areas, PVALB+ interneurons make up to 50% of the interneuronal population and target up to 200 pyramidal cells per single cell. Even small changes in interneuronal numbers or functionality can interfere with the proper orchestra of neuronal function in distinct cortical areas [25, 58, 59]. Impairments of GABAergic interneurons have been related to preterm birth brain injury and oxidative stress (OS). These impairments include the observed maldevelopment of cortical PVALB+ GABAergic interneurons [22, 23, 26]. Maturational and functional dysregulation of cortical interneurons, as observed by decreased density of PVALB+ interneurons, impaired GABA homeostasis, alteration of PNN formation, and delayed synaptogenesis in this study, could be held responsible for behavioral abnormalities as previously observed in this oxidative stress preterm birth brain injury model [30, 60] and also in neonatal patients [61, 62].

All PVALB+ interneurons are fast-spiking interneurons [63]. Due to their fast cellular metabolism and increased mitochondrial function, PVALB+ cells are particularly vulnerable to OS [35]. They have the highest myelination proportion of all GABAergic interneurons with more than 97% of axonal myelination [64], ensuring fast signal transduction. During development, most of the somata and proximal dendrites of cortical PVALB+ cells are covered by aggregated lattice-like structures of extracellular matrix called perineuronal nets (PNNs). In the rodent brain, the formation of PNNs occurs during brain maturation and coincides with synaptogenesis and synaptic refinement as well as myelination [37]. A pronounced function of PNNs is to maintain the ability of high firing frequencies in PVALB+ fast-spiking interneurons. It is discussed that PNNs protect PVALB+ cells from environmental and OS damage during critical periods of brain plasticity and brain development [65, 66]. However, OS itself may also impair PNN formation, as observed in this study. PNN removal around PVALB+ interneurons decreases the secretion of GABA and redirects them to a juvenile-like, less mature state [36, 67]. In accordance with this, the observed decreased GABA intensity after exposure to neonatal hyperoxia/OS at P30 lays in close relation to impaired PNN formation. Moreover, alterations of GAD67 expression, GABA intensity, and PNN formation strengthen the idea of maturational deficits of PVALB+ interneurons as observed by increased expression of *Gabra5* at the same time point, pointing towards a more immature developmental state. Gene expression analysis of other *Gabra* subtypes showed unaffected results and hence did not reveal signs of maturational deficits of GABAergic interneurons.

PNN formation starts during synaptogenesis and myelination, and cortical PNNs first appear around P14 [37]. As previously described, myelin formation is delayed in this neonatal OS model and may impair interneuronal development in rodents [13, 30] and in humans [22]. As indicated by this study, synaptogenesis can also be hampered by neonatal OS exposure, since expression of *Syn1*, *Syn2*, and *Syp* was decreased.

So, neonatal OS could have disturbed oxidative sensitive signaling indispensable for PNN formation. OS induced protein nitration, as observed by nitrotyrosine Western blot, which has been reported to alter protein function [34]. Protein nitration could therefore have acted as secondary hit after prior oxidative stress injury in brain development, and it can be assumed that protein nitration also represents a causative factor for decreased PNN formation in our neonatal OS mice. Additionally, PNNs are known to promote interneuron maturation synaptic/network stability and protection against oxidative stress [68].

A pathway that is also important for regulation of synaptogenesis is represented by the PI3K pathway [54, 69]. In theory, the previously defined reduction in glial cell-derived neurotrophic factor (GDNF) expression in cortical samples [30] can serve as an explanation for the inhibited PI3K signaling obtained in mice after exposure to neonatal hyperoxia/OS [70]. Hence, the decrease in PI3K-activity at ages P7 and P9 can be regarded as a cause or contributing factor leading to decreased *Syn1*, *Syn2*, and *Syp* expression. PDK1, the key downstream effector of PI3K signaling, positively regulates the survival of developing cortical interneurons [56]; however, increased apoptotic cell death of GABAergic interneurons was not observed in this model [30]. OS has been shown to possibly activate PI3K signaling [71], and agents inducing oxidative damage can regulate PI3K activity [72], which stands in contrast to our results of lower PI3K and pAKT expression after OS exposure. However, PI3K signaling in our neonatal OS model may have been affected secondarily by the decrease in GDNF expression.

Furthermore, lower GDNF expression after neonatal OS can also be responsible for the reduction of synapse formation, since GDNF strongly promotes synaptogenesis via synapsins [73]. Various synaptic functions are regulated by synapsins, including the formation of presynaptic terminals, regulation of the vesicle reserve pool at presynaptic terminals, synaptogenesis, elongation of axons, synaptic vesicle docking, and neurotransmitter release [48, 50, 74]. In theory, the delay in synapse formation can diminish GABA transmission at early time points of brain development, which is of high relevance since GABA signaling plays a central role in regulating cortical development [75]. Accordingly, decreased GABA activity can impair white matter development and neuronal development [76, 77] and might in our animals exposed to neonatal OS contribute or enhance OS-induced brain injury.

In general, impairments of cortical PVALB+ interneurons and PNNs play a role for psychiatric symptoms and behavioral deficits in several OS injury models [68]. The changes in PVALB+ interneuron maturation and in functional properties found in our neonatal OS model are likely to contribute to motor, social, and learning behavior that were previously determined in this model [30, 60].

## 5. Conclusion

Due to our data, neonatal OS interferes with cortical synaptogenesis as well as with PVALB+ interneuron formation and maturation. Apart from direct OS injury, neonatal OS exposure can lead to secondary hit mechanisms, as observed by decreased GABA intensity of PVLAB+ interneurons at later ages. Therefore, neonatal brain injuries might also affect developmental processes that occur after the primary injury. Additionally, complex behavioral alterations cannot be traced down to only one cell type in the case of an environmental OS injury model, in which the whole brain is exposed to an injurious stimulus and various brain cell types will be affected. The advantage of such scientific model, however, can be seen in the translational character in order to mimic the environmental challenges of preterm infants and correlate them to resulting cellular pathologies and to behavioral problems. Our study supports the notion that premature birth is leading to increased OS, which causes interneuronal deficits and contributes to the clinical phenotype often found in preterm infants. Prevention of OS in GABAergic interneurons may therefore serve as a target for neuroprotective strategies.

## Figures and Tables

**Table 1:** Oligonucleotides.

Gene		Forward primer	Reverse primer
*Gabra1*	NM_010250.5	CAGAAGATGGGTCACGTTTAAACC	GGAAGTGAGTCGTCATAACCACAT
*Gabra3*	NM_008067.4	GGGACAGAGATAATCCGGTCTAGTAC	GGTCTGGATGACAAAGTAGCCAAT
*Gabra4*	NM_001359041.1	GAAGTCAGTGGAGGTGCCAAA	CTGAGGTGGAAGTAAACCGTCAT
*Gabra5*	NM_176942.4	CAGCACCAGCACAGGTGAAT	GGAAGGTAGGTCTGGATGACAAA
*Hprt*	NM_013556.2	TGCTCGAGATGTCATGAAGG	TATGTCCCCCGTTGACTGAT
*Syn1*	NM_013680.4	ATCTTGTGGCTCATGCCAAT	ACACTGGGGATTCCAGCATA
*Syn2*	NM_001111015.1	CTACCCCAATCACCGAGAGA	ATACTTGGCGTCGATGAAGG
*Syp*	NM_009305.2	TGACTTTTTCCCCCTTCCTT	TTGGCTGACTGGTCCTCTCT
*Syt1*	NM_001252341.1	TGACCTGCTGCTTCTGTGTC	TGGGCTCCTCCTTTTCTTCT
*Syt2*	NM_009307.4	TACAAGGCGGAGAGAAGGAA	CTGCATCAGGTGGATCTTCA

## Data Availability

The data that support the findings of this study are available from the corresponding author upon request.

## References

[1] Trayhurn P. (2019). Oxygen—a critical, but overlooked, nutrient. *Frontiers in Nutrition*.

[2] Torres-Cuevas I., Parra-Llorca A., Sánchez-Illana A. (2017). Oxygen and oxidative stress in the perinatal period. *Redox Biology*.

[3] Castillo A., Sola A., Baquero H. (2008). Pulse oxygen saturation levels and arterial oxygen tension values in newborns receiving oxygen therapy in the neonatal intensive care unit: is 85% to 93% an acceptable range?. *Pediatrics*.

[4] Ikonomidou C., Kaindl A. M. (2011). Neuronal death and oxidative stress in the developing brain. *Antioxidants & Redox Signaling*.

[5] Malik S., Vinukonda G., Vose L. R. (2013). Neurogenesis continues in the third trimester of pregnancy and is suppressed by premature birth. *The Journal of Neuroscience*.

[6] Reich B., Hoeber D., Bendix I., Felderhoff-Mueser U. (2017). Hyperoxia and the immature brain. *Developmental Neuroscience*.

[7] Obst S., Herz J., Alejandre Alcazar M. A. (2022). Perinatal hyperoxia and developmental consequences on the lung-brain axis. *Oxidative Medicine and Cellular Longevity*.

[8] Lázár R., Orvos H., Szőllősi R., Varga I. S. (2015). The quality of the antioxidant defence system in term and preterm twin neonates. *Redox Report*.

[9] Chen X., Guo C., Kong J. (2012). Oxidative stress in neurodegenerative diseases. *Neural Regeneration Research*.

[10] Rodrigues C. A. V., Diogo M. M., da Silva C. L., Cabral J. M. S. (2010). Hypoxia enhances proliferation of mouse embryonic stem cell-derived neural stem cells. *Biotechnology and Bioengineering*.

[11] Fathollahipour S., Patil P. S., Leipzig N. D. (2019). Oxygen regulation in development: lessons from embryogenesis towards tissue engineering. *Cells, Tissues, Organs*.

[12] Zhang K., Zhu L., Fan M. (2011). Oxygen, a key factor regulating cell behavior during neurogenesis and cerebral diseases. *Frontiers in Molecular Neuroscience*.

[13] Schmitz T., Ritter J., Mueller S., Felderhoff-Mueser U., Chew L.-J., Gallo V. (2011). Cellular changes underlying hyperoxia-induced delay of white matter development. *Journal of Neuroscience: The Official Journal of the Society for Neuroscience*.

[14] Serdar M., Herz J., Kempe K. (2018). Protection of oligodendrocytes through neuronal overexpression of the small GTPase Ras in hyperoxia-induced neonatal brain injury. *Frontiers in Neurology*.

[15] Sakurai T., Gamo N. J. (2019). Cognitive functions associated with developing prefrontal cortex during adolescence and developmental neuropsychiatric disorders. *Neurobiology of Disease*.

[16] Rahman M. M., Islam M. R., Islam M. T. (2022). Stem cell transplantation therapy and neurological disorders: current status and future perspectives. *Biology*.

[17] Basu S. K., Pradhan S., du Plessis A. J., Ben-Ari Y., Limperopoulos C. (2021). GABA and glutamate in the preterm neonatal brain: in-vivo measurement by magnetic resonance spectroscopy. *NeuroImage*.

[18] Achim K., Salminen M., Partanen J. (2014). Mechanisms regulating GABAergic neuron development. *Cellular and Molecular Life Sciences*.

[19] Arshad A., Vose L. R., Vinukonda G., Hu F., Yoshikawa K., Csiszar A. (2016). Extended production of cortical interneurons into the third trimester of human gestation. *Cerebral Cortex*.

[20] Lussier S. J., Stevens H. E. (2016). Delays in GABAergic interneuron development and behavioral inhibition after prenatal stress. *Developmental Neurobiology*.

[21] Vasistha N. A., Pardo-Navarro M., Gasthaus J. (2020). Maternal inflammation has a profound effect on cortical interneuron development in a stage and subtype-specific manner. *Psychiatry*.

[22] Stolp H. B., Fleiss B., Arai Y. (2019). Interneuron development is disrupted in preterm brains with diffuse white matter injury: observations in mouse and human. *Frontiers in Physiology*.

[23] Panda S., Dohare P., Jain S. (2018). Estrogen treatment reverses prematurity-induced disruption in cortical interneuron population. *Journal of Neuroscience: The Official Journal of the Society for Neuroscience*.

[24] Rudy B., Fishell G., Lee S., Hjerling-Leffler J. (2011). Three groups of interneurons account for nearly 100% of neocortical GABAergic neurons. *Developmental Neurobiology*.

[25] Tremblay R., Lee S., Rudy B. (2016). GABAergic interneurons in the neocortex: from cellular properties to circuits. *Neuron*.

[26] Cabungcal J.-H., Steullet P., Kraftsik R., Cuenod M., Do K. Q. (2013). Early-life insults impair parvalbumin interneurons via oxidative stress: reversal by _N_ -acetylcysteine. *Psychiatry*.

[27] Marín O. (2012). Interneuron dysfunction in psychiatric disorders. *Reviews in the Neurosciences*.

[28] Hashemi E., Ariza J., Rogers H., Noctor S. C., Martínez-Cerdeño V. (2016). The number of parvalbumin-expressing interneurons is decreased in the prefrontal cortex in autism. *Cerebral Cortex*.

[29] Felderhoff-Mueser U., Bittigau P., Sifringer M. (2004). Oxygen causes cell death in the developing brain. *Neurobiology of Disease*.

[30] Scheuer T., dem Brinke E. A., Grosser S. (2021). Reduction of cortical parvalbumin-expressing GABAergic interneurons in a rodent hyperoxia model of preterm birth brain injury with deficits in social behavior and cognition. *Development*.

[31] Livak K. J., Schmittgen T. D. (2001). Analysis of relative gene expression data using real-time quantitative PCR and the 2− *ΔΔ*CT method. *Methods*.

[32] Scheuer T., Sharkovska Y., Tarabykin V. (2017). Neonatal hyperoxia perturbs neuronal development in the cerebellum. *Molecular Neurobiology*.

[33] Endesfelder S., Zaak I., Weichelt U., Bührer C., Schmitz T. (2014). Caffeine protects neuronal cells against injury caused by hyperoxia in the immature brain. *Free Radical Biology and Medicine*.

[34] Abdelmegeed M. A., Song B.-J. (2014). Functional roles of protein nitration in acute and chronic liver diseases. *Oxidative Medicine and Cellular Longevity*.

[35] Wen T. H., Binder D. K., Ethell I. M., Razak K. A. (2018). The perineuronal “safety” net? Perineuronal net abnormalities in neurological disorders. *Frontiers in Molecular Neuroscience*.

[36] Wingert J. C., Sorg B. A. (2021). Impact of perineuronal nets on electrophysiology of parvalbumin interneurons, principal neurons, and brain oscillations: a review. *Frontiers in Synaptic Neuroscience*.

[37] Brückner G., Grosche J., Schmidt S. (2000). Postnatal development of perineuronal nets in wild-type mice and in a mutant deficient in tenascin-R. *The Journal of Comparative Neurology*.

[38] Härtig W., Brauer K., Brückner G. (1992). Wisteria floribunda agglutinin-labelled nets surround parvalbumin-containing neurons. *Neuroreport*.

[39] Le Magueresse C., Monyer H. (2013). GABAergic interneurons shape the functional maturation of the cortex. *Neuron*.

[40] Lee S.-E., Lee Y., Lee G. H. (2019). The regulation of glutamic acid decarboxylases in GABA neurotransmission in the brain. *Pharmaceutical Research*.

[41] Rimvall K., Sheikh S. N., Martin D. L. (1993). Effects of increased *γ*-aminobutyric acid levels on GAD67 protein and mRNA levels in rat cerebral cortex. *Journal of Neurochemistry*.

[42] Kajita Y., Mushiake H. (2021). Heterogeneous GAD65 expression in subtypes of GABAergic neurons across layers of the cerebral cortex and hippocampus. *Behavioral Neuroscience*.

[43] Huttenlocher P. R., Dabholkar A. S. (1997). Regional differences in synaptogenesis in human cerebral cortex. *The Journal of Comparative Neurology*.

[44] Lammertink F., Vinkers C. H., Tataranno M. L., Benders M. J. N. L. (2021). Premature birth and developmental programming: mechanisms of resilience and vulnerability. *Psychiatry*.

[45] Curristin S. M., Cao A., Stewart W. B. (2002). Disrupted synaptic development in the hypoxic newborn brain. *Proceedings of the National Academy of Sciences of the United States of America*.

[46] Cobley J. N., Fiorello M. L., Bailey D. M. (2018). 13 reasons why the brain is susceptible to oxidative stress. *Redox biology*.

[47] Karson M. A., Tang A.-H., Milner T. A., Alger B. E. (2009). Synaptic cross talk between perisomatic-targeting interneuron classes expressing cholecystokinin and parvalbumin in hippocampus. *Journal of Neuroscience: The Official Journal of the Society for Neuroscience*.

[48] Feliciano P., Matos H., Andrade R., Bykhovskaia M. (2017). Synapsin II regulation of GABAergic synaptic transmission is dependent on interneuron subtype. *Journal of Neuroscience: The Official Journal of the Society for Neuroscience*.

[49] Bouhours B., Gjoni E., Kochubey O., Schneggenburger R. (2017). Synaptotagmin2 (Syt2) drives fast release redundantly with Syt1 at the output synapses of parvalbumin-expressing inhibitory neurons. *Journal of Neuroscience: The Official Journal of the Society for Neuroscience*.

[50] Forte N., Binda F., Contestabile A., Benfenati F., Baldelli P. (2020). Synapsin I synchronizes GABA release in distinct interneuron subpopulations. *Cerebral Cortex*.

[51] Song S.-H., Augustine G. J. (2015). Synapsin isoforms and synaptic vesicle trafficking. *Cell*.

[52] Gross C., Bassell G. J. (2014). Neuron-specific regulation of class I PI3K catalytic subunits and their dysfunction in brain disorders. *Frontiers in Molecular Neuroscience*.

[53] Jaworski J., Spangler S., Seeburg D. P., Hoogenraad C. C., Sheng M. (2005). Control of dendritic arborization by the phosphoinositide-3’-kinase-Akt-mammalian target of rapamycin pathway. *Journal of Neuroscience: The Official Journal of the Society for Neuroscience*.

[54] Cuesto G., Enriquez-Barreto L., Caramés C. (2011). Phosphoinositide-3-kinase activation controls synaptogenesis and spinogenesis in hippocampal neurons. *Journal of Neuroscience: The Official Journal of the Society for Neuroscience*.

[55] Sellers K. J., Erli F., Raval P., Watson I. A., Chen D., Srivastava D. P. (2015). Rapid modulation of synaptogenesis and spinogenesis by 17Î²-estradiol in primary cortical neurons. *Frontiers in Cellular Neuroscience*.

[56] Wei Y., Han X., Zhao C. (2020). PDK1 regulates the survival of the developing cortical interneurons. *Molecular Brain*.

[57] Wonders C. P., Anderson S. A. (2006). The origin and specification of cortical interneurons. *Reviews in the Neurosciences*.

[58] Ariza J., Rogers H., Hashemi E., Noctor S. C., Martínez-Cerdeño V. (2018). The number of chandelier and basket cells are differentially decreased in prefrontal cortex in autism. *Cerebral Cortex*.

[59] Nahar L., Delacroix B. M., Nam H. W. (2021). The role of parvalbumin interneurons in neurotransmitter balance and neurological disease. *Psychiatry*.

[60] Schmitz T., Endesfelder S., Reinert M.-C. (2012). Adolescent hyperactivity and impaired coordination after neonatal hyperoxia. *Experimental Neurology*.

[61] Arpi E., Ferrari F. (2013). Preterm birth and behaviour problems in infants and preschool-age children: a review of the recent literature. *Developmental Medicine & Child Neurology*.

[62] Johnson S., Wolke D., Hennessy E., Marlow N. (2011). Educational outcomes in extremely preterm children: neuropsychological correlates and predictors of attainment. *Developmental Neuropsychology*.

[63] Hu H., Gan J., Jonas P. (2014). Interneurons. Fast-spiking, parvalbumin^+^ GABAergic interneurons: from cellular design to microcircuit function. *Science*.

[64] Stedehouder J., Couey J. J., Brizee D. (2017). Fast-spiking parvalbumin interneurons are frequently myelinated in the cerebral cortex of mice and humans. *Cerebral Cortex*.

[65] Cabungcal J.-H., Steullet P., Morishita H. (2013). Perineuronal nets protect fast-spiking interneurons against oxidative stress. *Proceedings of the National Academy of Sciences*.

[66] Reichelt A. C., Hare D. J., Bussey T. J., Saksida L. M. (2019). Perineuronal nets: plasticity, protection, and therapeutic potential. *Trends in Neurosciences*.

[67] Lensjø K. K., Lepperød M. E., Dick G., Hafting T., Fyhn M. (2017). Removal of perineuronal nets unlocks juvenile plasticity through network mechanisms of decreased inhibition and increased gamma activity. *Journal of Neuroscience: The Official Journal of the Society for Neuroscience*.

[68] Steullet P., Cabungcal J.-H., Coyle J. (2017). Oxidative stress-driven parvalbumin interneuron impairment as a common mechanism in models of schizophrenia. *Molecular Psychiatry*.

[69] de Curtis I. (2014). Roles of Rac1 and Rac3 GTPases during the development of cortical and hippocampal GABAergic interneurons. *Frontiers in Cellular Neuroscience*.

[70] Neff F., Noelker C., Eggert K., Schlegel J. (2002). Signaling pathways mediate the neuroprotective effects of GDNF. *Annals of the New York Academy of Sciences*.

[71] Uranga R. M., Katz S., Salvador G. A. (2013). Enhanced phosphatidylinositol 3-kinase (PI3K)/Akt signaling has pleiotropic targets in hippocampal neurons exposed to iron-induced oxidative stress∗. *The Journal of Biological Chemistry*.

[72] Li K., Wu L., Chen Y. (2020). Cytotoxic and antiproliferative effects of *β*-mangostin on rat C6 glioma cells depend on oxidative stress induction via PI3K/AKT/mTOR pathway inhibition. *Drug Design, Development and Therapy*.

[73] Ledda F., Paratcha G., Sandoval-Guzmán T., Ibáñez C. F. (2007). GDNF and GFR*α*1 promote formation of neuronal synapses by ligand-induced cell adhesion. *Nature Neuroscience*.

[74] Mirza F. J., Zahid S. (2018). The role of synapsins in neurological disorders. *Neuroscience Bulletin*.

[75] Wang D. D., Kriegstein A. R. (2009). Defining the role of GABA in cortical development. *The Journal of Physiology*.

[76] Pudasaini S., Friedrich V., Bührer C., Endesfelder S., Scheuer T., Schmitz T. (2022). Postnatal myelination of the immature rat cingulum is regulated by GABAB receptor activity. *Developmental Neurobiology*.

[77] Gustorff C., Scheuer T., Schmitz T., Bührer C., Endesfelder S. (2021). GABAB receptor-mediated impairment of intermediate progenitor maturation during postnatal hippocampal neurogenesis of newborn rats. *Frontiers in Cellular Neuroscience*.

